# The influence of psychological change factors of tennis training strategy using optimized recurrent neural network and artificial intelligence

**DOI:** 10.1016/j.heliyon.2024.e33273

**Published:** 2024-06-19

**Authors:** Yan Du, Yujia Xia, Lili Wang, Tiantian Zhang, Linlin Ju

**Affiliations:** a School of Sociology and Anthropology, Xiamen University, Xiamen, 361005, China; b Sports Department, Southwest University of Political Science and Law, Chongqing, 400031, China; c School of International Business & Management, Chongqing Institute of Foreign Studies, Chongqing, China; d College of Physical Education, East China University of Technology, Nanchang, China; e School of Marxism, Sichuan International Studies University, Chongqing, 400031, China; f College of Physical Education, China West Normal University, Nanchong, 637009, China

**Keywords:** Tennis training, Psychological changes, RNN, AI, Psychological regulation

## Abstract

Due to the specialization of tennis technical training, the teaching focus of tennis teaching has gradually shifted to the psychological skills training of tennis players. This work addresses the impact of psychological factors on tennis players' insufficient concentration in teaching and training on match results. It discusses the psychological changes' influencing factors in tennis training strategies and analyzes the current psychological changes that are easy to occur in tennis teaching. The Recurrent Neural Network (RNN) can simulate the human brain's information processing and learning process to establish models to study human psychological changes. To explore the influence of psychological changes on tennis training, artificial intelligence technology is combined to optimize the performance of RNN, and a prediction model of psychological distress in tennis training is constructed. Additionally, a questionnaire is applied to compare the sports state of tennis players before and after the psychological regulation intervention. The findings demonstrate that following psychological regulation, 73 % of players perform as usual, 20 % present exceptionally well, and 7 % do not perform as well as usual. These results indicate an improvement compared to previous performances, highlighting the efficacy of psychological regulation supported by optimized RNN under AI assistance. This study aims to foster a consistently positive psychological state among tennis players during daily training and competitions, ensuring that their competitive performance levels remain normal or even exceed their usual standards.

## 1 Introduction

The influence of psychological factors is crucial for athletes, with a positive mindset being essential for achieving favorable competition results. As a highly personalized sport, tennis demands exceptional psychological skills from its athletes. In a tennis match where athletes possess equal technical and tactical proficiency under ideal conditions, the outcome largely depends on the strength of their psychological resilience (Sieka World et al., 2021). Hence, the significance of robust psychological skills is self-evident [1]. Excessive psychological pressure can lead to mental depression and physical fatigue. Especially, during critical moments in major competitions, causing athletes to lose their technical advantages and directly impacting their performance outcomes [2]. Consequently, it is essential to study the psychological changes in tennis training strategies and adjust the players' psychological states in response to these factors.

Currently, AI, as new productivity, has significantly transformed the traditional sports industry. The AI technology application enables the acquisition of athletes' sports performance data through wearable devices to assist coaches in auxiliary training [3]. However, while RNN has widespread applications, it suffers from slow convergence speed and tends to fall into local minima [4]. In recent years, the government has successively introduced relevant policies to upgrade AI technology to the national strategic level. In the future, sports and AI can be more deeply integrated. This work focuses on optimizing RNN using AI technology and applying optimized RNN to investigate psychological factors influencing tennis training strategies. An improved agent genetic algorithm (GA) is proposed, employing group cooperation multi-agent GA to optimize the neural network.

In light of these challenges, this work examines the factors influencing psychological changes in tennis training strategies and analyzes the common psychological changes observed in tennis training.

## 2 Literature review

Athletes' psychological adaptability and on-the-spot control level are crucial components in competitive sports. Relevant scholars have extensively researched methods for regulating and controlling athletes' psychological states. Costa et al. [5] indicated that the content of sports psychology in China mainly involved athletes' self-confidence, motivation, emotion, attention, willingness, cognition, fatigue, and interpersonal relationships. Popovych, Blynova, Nosov, Zinchenko, and Kononenko [6] summarized athletes' psychological regulation methods as establishing psychological behavior programs, systematic simulation training, publicity and education, psychological speeches, and sports psychology expert's support network system. Cece, Guillet-Descas, Nicaise, Lienhart, and Martinent [7] addressed the application issues of sports psychology through Solution-Focused Brief Therapy, autonomous physiological coherence systems, and sprint strategy interventions. It encompassed mobile psychological adjustment vehicles, music therapy, and other forms. Moreno-Pérez et al. studied pre-competition mental regulation and control among college tennis players. Their research involved the literature review and questionnaires to investigate pre-competition mental regulation training methods. Based on the observed characteristics of pre-competition psychological changes, the study proposed several strategies: enhancing pre-competition psychological training, preparing tactical psychological readiness before competitions, and addressing various adverse psychological reactions encountered prior to competitions [8].

As scientists employ increasingly sophisticated techniques to investigate RNNs, recent research highlights various advancements. For instance, in exploring the application of artificial intelligence (AI) for analyzing tennis technical movements, Yanan et al. proposed that artificial neural networks (ANNs) could infer conclusions through implicit algorithms, bypassing the need to fully comprehend the technical rules of movements. This approach facilitated the construction of a knowledge base on biomechanics of technical movements in tennis. The integration of ANNs with the development of explicit rules derived from this knowledge base was anticipated to emerge as a significant trend in tennis technology diagnosis moving forward. Moreover, the application of AI in the analysis of movement technology can represent major progress in developing sports biomechanics [9]. Zappone, Di Renzo, Debbah, Lam, and Qian [10] designed an RNN module of an embedded AI processor. By customizing the design of the instruction set, input/output system, neuron computing unit, and storage system, the real-time and efficient deployment of the Long Short-Term Memory (LSTM) in the RNN was achieved. Jekauc et al. indicated that the RNN-based model achieved an accuracy of up to 68.9 %, surpassing or equaling human observers in several instances. Interestingly, both machine learning (ML) models and human observers demonstrated a shared proficiency in identifying negative emotional states more effectively, likely due to the clearer and more direct expression of these states [11].

To sum up, relevant scholars have some studies on the psychological regulation and RNN of competitive sports. Psychological prediction intervention on athletes has practical significance for improving the athletes' performance. The application of AI to RNN is a research hotspot currently. Combining the needs and advantages of both fields, this work explores the influence of psychological change factors on tennis training strategy using an optimized RNN assisted by AI. It implements a prediction model for psychological distress in tennis training. Moreover, before and after psychological regulation intervention, a questionnaire is employed to compare tennis players’ sports state, analyzing the strengths of psychological regulation strategies based on optimized RNN assisted by AI.

## 3 Research methodology

### 3.1 Psychological characteristics of players in tennis training

In sports competitions, the psychological changes of athletes greatly impact their performance, because they can directly affect the heartbeat, respiration, and muscle tension, thus determining the athletes' performance in the competition. In some high-level competitions, the technical level of athletes is almost the same, and the competition between psychological endurance and will quality is the key to athletes’ true competition [12]. Currently, most sports powers have strengthened the psychological guidance for athletes, and multiple countries have also set up special psychological expert groups to regulate athletes' psychology [13]. In sports competitions, athletes' psychological changes significantly impact their performance, affecting their heartbeat, respiration, and muscle tension, which in turn determine their competitive outcomes. In high-level competitions, where technical skills are almost equivalent, the competition often hinges on psychological endurance and willpower [12]. Currently, many leading sports nations have strengthened psychological guidance for athletes and have established special expert groups to regulate athletes' psychological states [13].

Emotion refers to the attitude experience of athletes to the competition, and it is a reaction to whether their needs are met. The negative emotional state in tennis players before the game is mainly manifested as excessive excitement or depression, exhibited through high mood, inattention, and disruptions in diet and sleep patterns well before the game [14]. The mood of tennis players also changes greatly. Tennis players' moods fluctuate significantly, as seen in their tendency to relax, hesitate, or become lenient when leading, and to collapse or surrender when in a stalemate or falling behind [15]. Post-game, negative emotions can persist and affect performance in subsequent matches [16].

**Fig. 1:**
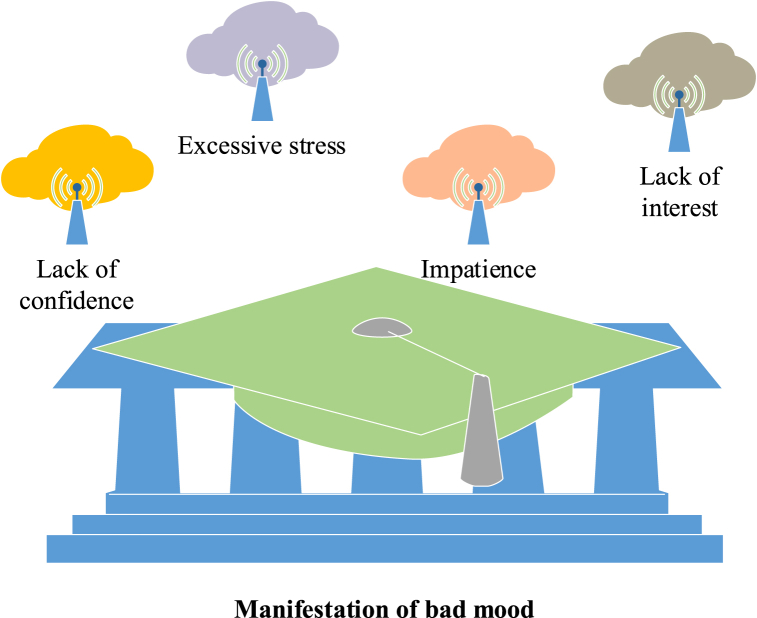
The manifestation of tennis players' bad mood.

Will quality in tennis players refers to the psychological process of consciously setting goals before competitions. This process enables players to regulate their actions according to their objectives, overcome difficulties, and achieve their goals [17]. Tennis, characterized by variable tactics, strong opposition, and prolonged rallies, demands strong will quality. Insufficient will quality leads to an inability to suppress fatigue. After intense running and hitting, physical strength depletes significantly. If players cannot overcome these reactions, they become tense and uncoordinated, thus affecting the serving and receiving quality [18]. These negative emotional states can be categorized into four primary conditions, as presented in Fig. 1.

**Fig. 2:**
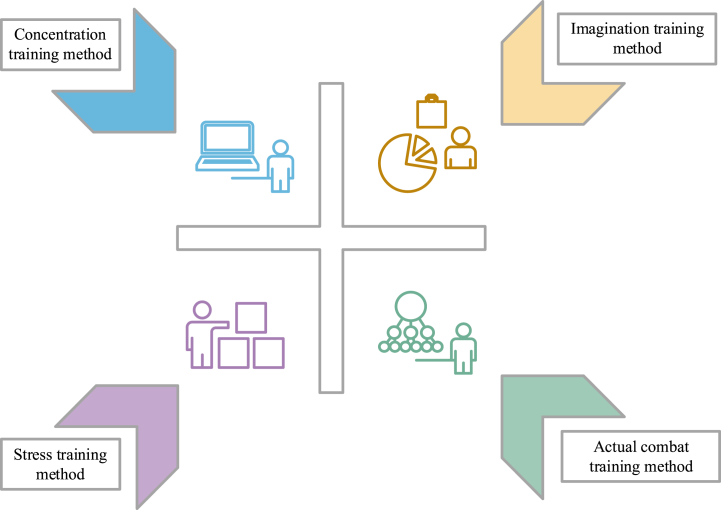
Methods of training athletes' psychological quality.

To avoid unhealthy psychological states during competition, athletes must undergo purposeful psychological training in addition to material selection, physical strength conditioning, and technical and tactical training. This approach allows athletes with different personalities to adjust their psychological functions through long-term sports training. They can mobilize psychological mechanisms at all levels, overcome timidity and nervousness, and enhance self-control. Ultimately, it maintains a competitive state characterized by energy and emotional stability, allowing them to perform their skills effectively [19]. Early psychological training offers multiple advantages and favorable conditions. Four primary methods are used to cultivate athletes' psychological qualities, as illustrated in Fig. 2.

Overcoming psychological barriers is the basis and key to improving tennis performance, integrating intelligence with physical strength. Hence, the training of psychological quality plays a pivotal part in the training of tennis players.

### 3.2 The use of AI combined with optimized RNN for psychological prediction

The recent research on AI chips has attracted increasing attention from academic and industrial circles, especially in the cloud of large-scale data and the terminal direction with special requirements. Chips and hardware systems specially designed for the AI direction are emerging endlessly [20]. This work plans to construct an RNN algorithm that can solve the gradient dependence problem, and improve the function of the existing AI chip. Thus, it expandes the application field of tennis players' action recognition and psychological prediction.

**Fig. 3:**
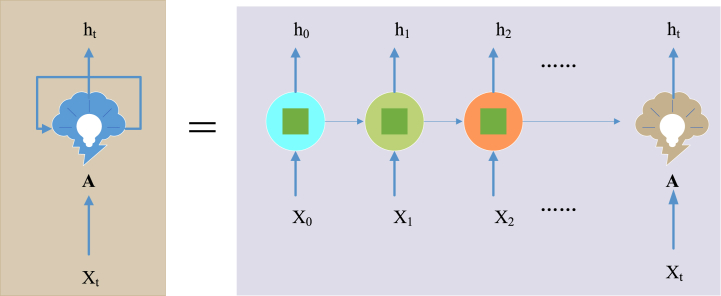
RNN structure.

RNN takes sequence data as input and recurses in the evolution direction of the sequence. All nodes form a closed loop by chain connection [21]. The research on RNN began in the 1980s–1990s and developed into a crucial deep learning (DL) algorithm at the beginning of the 21st century. Bidirectional RNN (Bi-RNN) and LSTM are common RNNs [22]. Fig. 3 displays the structure of RNN expanded based on time.

**Fig. 4:**
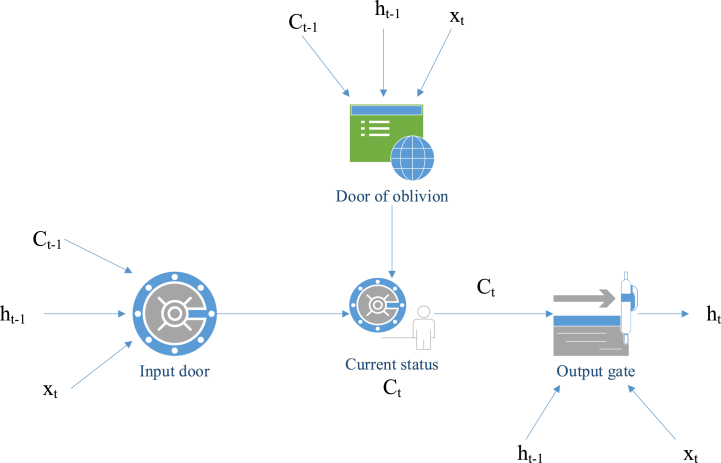
LSTM network structure.

LSTM is a special network structure with three "gates". It relies on the structure of some "gates" to make information selectively affect the state of each time in the neural network [23]. The "gate" structure is an operation that uses a sigmoid neural network and a bitwise multiplication. It is called a "gate" because the fully connected layer using sigmoid as the activation function outputs a value between 0 and 1, describing how much information the current input can pass through this structure. This structure functions analogously to a gate. When the gate is open, indicated by the sigmoid neural network layer's output being 1, all information can pass through. Conversely, when the gate is closed, with this layer's output being 0, no information can pass through [24]. Fig. 4 presents the schematic diagram of its unit structure.

The calculation of the forget gate in the LSTM algorithm is as follows:

(1) $$f_{t}=\sigma \left(W_{f}\cdot \left[h_{t-1},x_{t}\right]+b_{f}\right)$$

σ(x) represents the activation function; $W_{f}$ and $b_{f}$ refer to the weight and offset term of the forget gate; $h_{t-1}$ and $x_{t}$ denote the output and input vectors at times t-1and t [25].

Next, it is to determine the new information stored in the cell state. This process includes two parts. The first step is to take $h_{t-1}$ and $x_{t}$ as inputs in the σ layer of the input gate, and decide to update the value $i_{t}$. The second step is to set up a new candidate value vector $\bar{C_{t}}$ in the tanh layer, and $\bar{C_{t}}$ is added to the state [26]. The calculation process reads:

(2) $$i_{t}=\sigma \left(W_{c}\cdot \left[h_{t-1},x_{t}\right]+b_{c}\right)$$

(3) $$\bar{C_{t}}=\tanh\;\left(W_{c}\cdot \left[h_{t-1},x_{t}\right]+b_{c}\right)$$

$b_{c}$ and $W_{c}$ represent offset term and weight of the input gate. According to the changes in the situation, the historical information that has little impact on the existing distribution characteristics is discarded. This step is to update the cell status [24]. The calculation is expressed in equation (4):

(4) $$C_{t}=f_{t}\cdot C_{t-1}+i_{t}\cdot \bar{C_{t}}$$

Finally, the value to be output is determined, and the output value is based on the current cell state. Step 1, $h_{t-1}$ and $x_{t}$ are input, and the part $o_{t}$ to be output through the σ layer is decided. Step 2, a value between −1 and 1 is obtained for the cell state through the tanh layer, and multiply it with the output of the σ layer to output the desired value [27]. The calculation can be written as equations (5), (6):

(5) $$o_{t}=\sigma \left(W_{0}\cdot \left[h_{t-1},x_{t}\right]+b_{0}\right)$$

(6) $$h_{t}=o_{t}\cdot \tanh\;\left(C_{t}\right)$$

$W_{0}$ and $b_{0}$ are the weight and offset term of the output gate.

The training algorithm of LSTM-RNN adopts the backpropagation algorithm, which includes several steps. First, the output values of each neuron are calculated forward according to equations (1), (2), (3), (4), (5), (6), namely the values of the five vectors ft, it, Ct, ot, and ht. Second, the error term δ for each neuron is calculated backward. Similar to RNN, the error term propagates in two directions. One is the backward propagation through time, and the other is the propagation to the previous layer. Third, based on the error terms, an optimization algorithm is used to adjust the model parameters by calculating the gradients of each weight, aiming to approach the optimization objective of the prediction results. Lastly, the model is trained through the iterative process described above until achieving the desired optimization objective, thus establishing an LSTM-RNN prediction model that meets the error requirements.

**Fig. 5:**
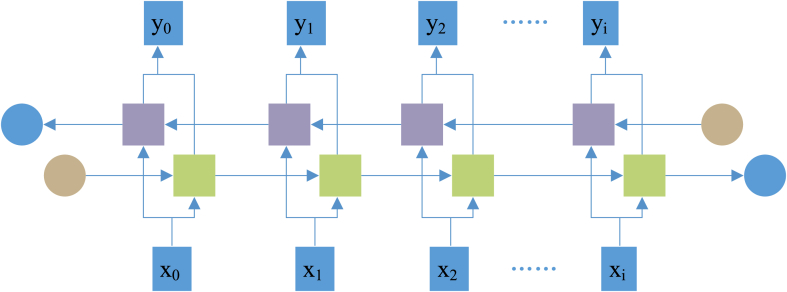
Bi-RNN network structure.

In classical RNN, the state transmission is one-way from front to back. However, in some problems, the output at the current time is related to the previous and subsequent states, requiring the use of Bi-RNN to solve this problem. Bi-RNN is composed of two unidirectional RNNs superposed together, and these two RNNs’ state determines the output. At each time t, the input is provided to the two RNNs in opposite directions simultaneously, while the output is determined by the two unidirectional RNNs [28]. Fig. 5 depicts its structure.

**Fig. 6:**
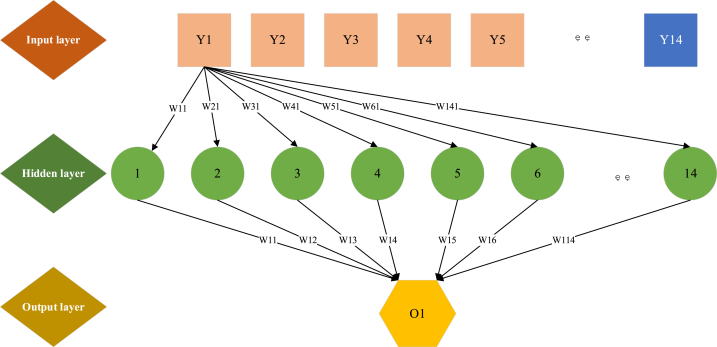
Optimized RNN structure.

Based on the aforementioned RNN optimization structure, this work utilizes the probabilistic adaptive iterative optimization ability of multi-agent GA to identify the chromosome with the highest probability for its fitness function. Notably, GA's global optimization proficiency operates independently of gradient information. The approach integrates GA to fine-tune the weight thresholds of RNN, broadening the search scope beyond RNN's initial random weight thresholds. This process effectively narrows the algorithm's search space, thus reducing computational complexity. By synergizing these algorithms, their respective strengths are leveraged synergistically. The optimized RNN demonstrates enhanced learning speed and significantly improved network approximation capabilities throughout the training process, bolstering network generalization. Fig. 6 displays its layered structure.

There are 14 neurons in the input layer, with Y_j_ representing the input data of the *j*th node in the output layer. Similarly, the hidden layer comprises 14 neurons, and the output layer contains a single neuron. The mental health status is derived through a fuzzy comprehensive evaluation. The output of the kth node in the network's output layer is denoted by O. The connection weight from the second layer of neuron i to the first layer of neuron j is w_ij_. The connection weight value from the third layer neuron k to the second layer neuron i is w_ki_.

The whole operation process is classified into two propagation directions: forward propagation and backpropagation. Forward propagation refers to the network fitting data from input to output, but only the trained network can work. Network training involves backpropagation, where the error between the model's output and the expected output is fed back to adjust the weight and threshold of each neuron adaptively. This enables the trained neural network to fit the data well.

The optimized RNN assisted by AI can effectively avoid the problem of gradient vanishing, solve the data dependency in long-term learning, and maintain the gradient influence in the linear region of geometric functions. To verify the proposed model's performance advantages, it is compared with a logistic regression (LR) prediction model. Meanwhile, the psychological status of college tennis players is analyzed, and the factors influencing the psychological changes of tennis players' training strategies are summarized.

## 4 Experimental design and performance evaluation

### 4.1 Datasets collection

Using a random cluster sampling method, 50 tennis athletes participating in college sports competitions are selected as the research subjects. The selection criteria are as follows: (1) training duration ≥1 year; (2) college students in grades 1–3; (3) voluntary participation in the study and good cooperation. Data collection primarily involves observing the athletes' performances on the court or during training sessions. The observation team consists of 5 coaches who simultaneously observe both groups. Based on observations of athletes' movements and emotions, conclusions are drawn regarding the accuracy of RNN predictions, enabling targeted psychological control interventions. Additionally, no abnormal conditions are observed during the observation process. Participants are divided into Group A (psychological regulation) and Group B (non-psychological regulation), with 25 individuals per group. Group B completes regular pre-competition training arrangements, while Group A conducts psychological regulation interventions on this basis, mainly employing the following methods for psychological regulation.

(1) Relaxation training: Before skills training, high-energy music is chosen to create a relaxed and awakening atmosphere under musical background, awakening the body, uplifting emotions, and focusing attention. After skills training, upbeat music is played for stretching exercises under a musical background to relieve physical fatigue and mental tension. Before bedtime, soothing music is played for deep slow abdominal breathing and meditation under a musical background, gradually inducing a relaxation state for the body and mind. Relaxation training lasts 10–15 min/session, 3 times/day. Weekly recreational team competitions, such as basketball and tug-of-war matches, are organized to provide psychological relaxation for athletes during physical exercise.
(2) Imagery training: Through one-on-one communication, athletes are helped to analyze their strengths and weaknesses in daily training and competitions. Deficiencies are supplemented with guidance, encouraging athletes to utilize their strengths. Athletes are encouraged to simulate sports states through imagination, forming competitive effects in their minds, thus promoting the establishment of optimal competitive states. Athletes fully understand and address their shortcomings or existing issues, finding solutions. This method is primarily used to regulate mood states, psychological fatigue, lack of confidence, anxiety, and irritability.
(3) Goal setting: Athletes are required to recall and summarize daily practice content and technical points before bedtime according to daily and weekly training plans and completion, analyzing and summarizing their progress and shortcomings. Moreover, they need to set short-term training goals (daily and weekly goals), urge themselves to complete training tasks, and reasonably arrange their daily lives under the completion of training content during training periods.
(4) Targeted interviews: Weekly psychological assessments are performed, and one-on-one interviews are conducted for athletes with more obvious psychological abnormalities. Targeted guidance and encouragement are provided to help alleviate negative emotions, lasting 20–30 min/session, once a week. The personnel situation is exhibited in Table 1:

**Table 1:** Basic information of subjects.

Group	Group A	Group B
Male	13 students	19 students
Female	12 students	6 students
Age	18∼23	18∼24
Years of exercise	1∼6 years	1∼5 years

Psychological regulation is primarily achieved through self-suggestion and improving transparency. The practice has proved that targeted self-suggestion can stabilize emotions, eliminate external interference, and mitigate the adverse effects of psychological state. The concept of "downplaying importance" involves helping athletes reduce their excessive focus on game outcomes and their concern about evaluations. This approach also aims to influence the reactions of those who provide social support to athletes, encouraging a more balanced perspective.

### 4.2 Experimental environment

Statistical Product and Service Solutions software is adopted to implement an RNN prediction model assisted by AI. The training set is input to allow the model to learn autonomously and discern the internal patterns within the data. The training parameters are defined as follows. There are 2 hidden layers, the training type is batch training, and the maximum training time is 15 min. In this experiment, the dataset is divided into two equally sized subsets, and the classifier undergoes two training sessions. In the first session, one subset serves as the training set while the other is used as the test set. In the second session, the training and test sets are exchanged. The modeling software employs random stratification, with 70 % of the samples selected as the training set for the RNN model's self-learning and training. The remaining 30 % of the samples constitute the test set, used to evaluate the model's accuracy and validate the established model's prediction accuracy. Statistically significant factors identified through univariate analysis are used as input layer neurons, while psychological distress indicators are designated as output layer neurons.

### 4.3 Parameters setting

AI supports the enhancement and broad application of RNN models through various means. For instance, AI techniques optimize the hyperparameter selection of RNNs, discovering the optimal model structure and parameter configuration through automated ML techniques. DL frameworks and tools also simplify the design, training, and deployment processes of RNN models.

The RNN model assisted by AI is configured with approximately 70 % of the samples designated as the training set and about 30 % as the test set. The hidden layer consists of two layers. The training type is batch training, and training is terminated when the error does not decrease over a continuous step. The maximum training time is set to 15 min. The prediction accuracy calculation follows the same method as the LR model, using 0.5 as the cut-off value. Thus, observations with a prediction probability ≥0.5, or <0.5 are considered positive and negative events.

### 4.4 Performance evaluation

**Fig. 7:**
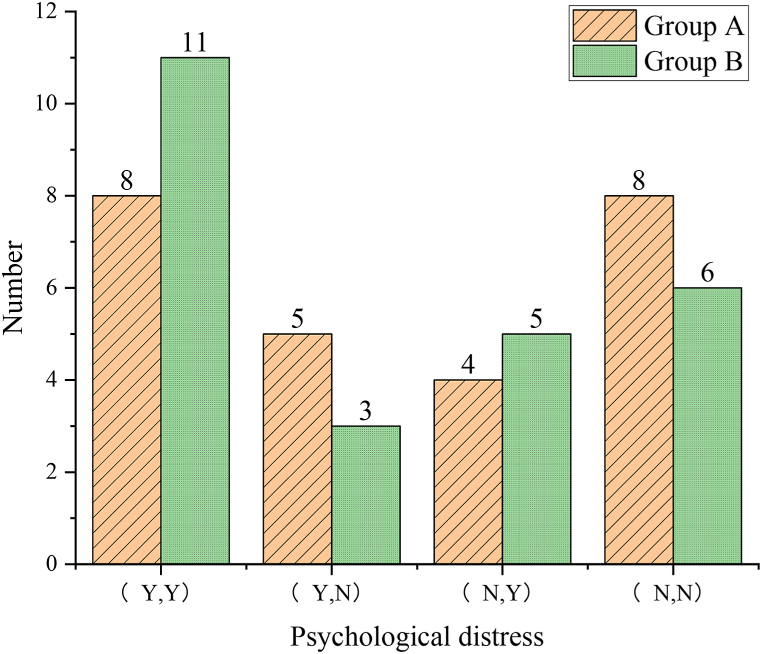
Prediction results of athletes' psychological conditions.

According to the psychological prediction results based on optimized RNN assisted by AI, the situation is classified into actual measurements and predictions. The accuracy of psychological prediction for Group A athletes is 84.56 %, and for Group B athletes, it is 84.02 %, both exceeding 80 %. The specific situation is revealed in Fig. 7.

**Fig. 8:**
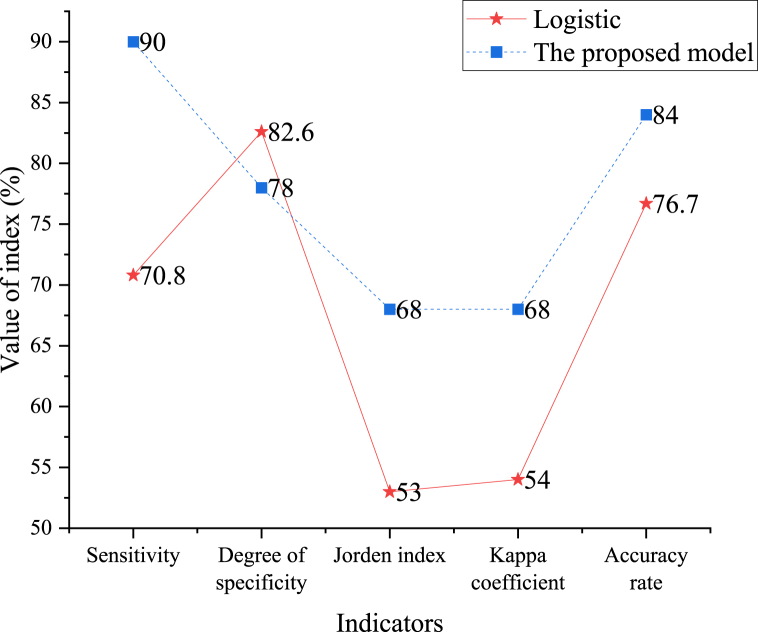
Comparison of indicators of two prediction models.

The relevant indicators of the LR prediction model are compared with those of the RNN model assisted by AI. The results reveal that the RNN model is superior to the LR model except for its specificity, showing a good prediction effect. Fig. 8 displays the comparison results of the main performance indicators of the two prediction models.

**Fig. 9:**
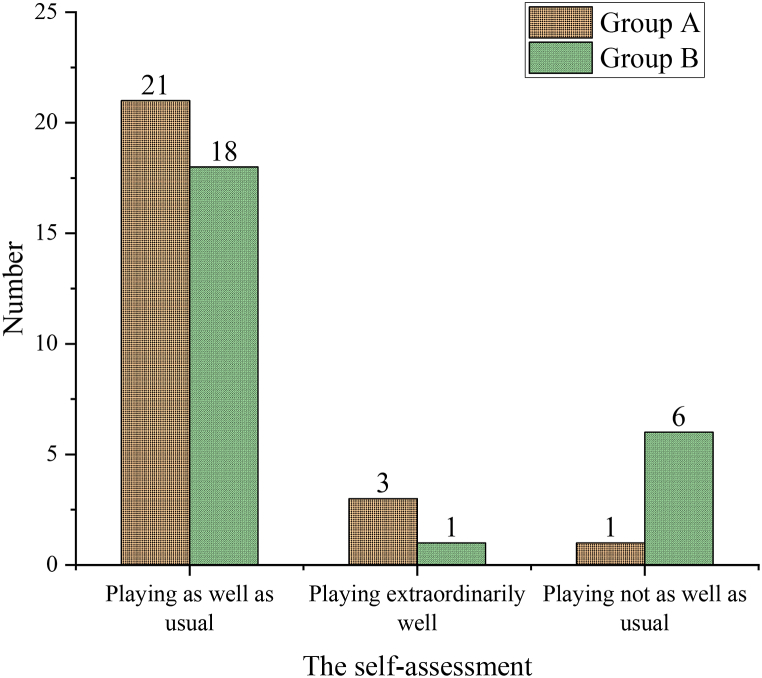
Self-assessment of the two groups of athletes.

Athletes with poor psychological states are identified for psychological regulation and their self-assessments are categorized as “playing as well as usual,” “playing extraordinarily well,” and “playing not as well as usual.” The results demonstrate that: the number of athletes in Group A who rate themselves as playing extraordinarily well, as well as usual, and not as well as usual is 3, 21, and 1, accounting for 12 %, 84 %, and 4 %, respectively. In Group B, the numbers are 1, 18, and 6, accounting for 4 %, 72 %, and 24 % respectively. The rate of Group A's self-assessment as “not as well as usual” is significantly lower than that of Group B. This indicates that timely identification of athletes' psychological states and subsequent psychological regulation can ensure normal performance in competition, and even foster extraordinary performance. Fig. 9 provides a detailed breakdown.

Athletes' psychological changes involve multifactorial interaction and mutual adjustment, where each factor's role and adjustment do not exhibit simple linear relationship. Hence, the traditional linear mixed model often has some limitations when dealing with data about human psychological changes. As a mathematical modeling method that simulates the functioning of biological neurons in the human brain, AI-assisted RNN can handle complex problems by simulating neuron interactions. In RNN, the hidden layer receives the data of the input layer for calculation and output, and the output result is compared with target values. If discrepancies arise, errors are backpropagated to adjust the weights of connections between neurons. Through constant learning and adjustment, the model achieves higher classification accuracy. Therefore, the complex nonlinear relationship between independent and response variables and the potential interaction among variables can be identified to achieve better prediction and discrimination functions. The comparison analysis of experimental data demonstrates that the AI-assisted optimized neural network effectively contributes to identifying tennis players' psychology. In addressing the occurrence of negative emotions, this study outlines appropriate psychological adjustment strategies. 1. Praise and encouragement: Instead of indiscriminate criticism when athletes fail to meet expected performance standards, coaches should offer more words of encouragement and praise. This approach helps athletes maintain psychological stability and resilience. 2. Attention diversion: During tennis training and competition, if athletes fall behind in scoring, coaches should redirect their attention towards more positive aspects. This method enables athletes to maintain emotional stability, focus on the game, and execute their skills and tactics effectively. These intervention strategies are designed to optimize athletes' psychological well-being and enhance their performance in training and competitive contexts.

Table 2 shows no statistically significant difference in the scores of various dimensions of the ABQ between the two groups (P > 0.05). Following the intervention, the scores of all dimensions of the ABQ in Group A are markedly decreased (P < 0.05), while there is no significant change in Group B (P > 0.05). The inter-group comparison shows a statistically significant difference (P < 0.05). This demonstrates that psychological intervention has a positive impact on psychological regulation, and that negative psychological factors such as sports negativity have a remarkable influence on tennis training.

**Table 2:** Comparison of ABQ scores between two groups before and after intervention.

Group	Negative evaluation of sports	Emotional/physical exhaustion	Reduced sense of achievement
Group A (n = 25)			
Before intervention After intervention	11.82 ± 2.67	12.33 ± 2.64	12.84 ± 2.43
After intervention	9.03 ± 1.95*	10.15 ± 1.67*	11.02 ± 1.88*
t	3.268	2.703	2.294
p	0.003	0.012	0.029
Group B (n = 25)			
Before intervention	11.67 ± 2.64	12.17 ± 2.56	12.76 ± 2.41
After intervention	10.93 ± 2.61	12.02 ± 2.47	12.55 ± 2.32
t	0.772	0.163	0.243
p	0.447	0.871	0.810

### 4.5 Discussion

This work primarily focuses on the early prediction of athletes' psychological states to facilitate timely adjustments during pre-competition, competition, and post-competition phases. Additionally, this work optimizes RNN with AI, comparing its performance with that of a LR prediction model. The results indicate that all indices except specificity are higher than those of the LR model. This approach can be widely applied to recognize and predict the psychological states of competitive athletes, thereby assisting students in overcoming anxiety during competitions and reducing the onset of sports fatigue. Consequently, it can lay a solid foundation for the comprehensive enhancement of psychological resilience.

## 5 Conclusion

The future development trend in the psychological regulation of tennis players involves establishing explicit rules by developing a knowledge base of tennis technology and analyzing technical movements with AI-assisted RNN. The application of AI in action technology analysis represents significant progress in sports biomechanics. Moreover, this system can be easily replicated and serve as a tool for training and cultivating other sports scientists, coaches, and athletes. The research results show that the stress of competitive sports, athletes' psychological reactions to the strength of their opponents before the competition, their level of self-confidence during the competition, their thoughts on the competition results after the competition, and their adaptability to the external environment greatly impact athletes' psychological mood. Therefore, it is suggested that college tennis coaches conduct comprehensive, complete, and systematic pre-competition psychological training for athletes based on their actual situation and regular technical training. It is essential to eliminate the negative emotions from multiple aspects before the competition, improve athletes' psychological endurance, establish a positive emotional state before competitions, and ultimately enhance the players’ competition ability and performance.

Due to the short research period and substantial workload of this work, there are some limitations in applying AI-assisted RNN analysis technology to analyze the impact factors of psychological changes in tennis training strategies. Future research can be carried out from the following two aspects: (1) Investigating how the factors influencing psychological changes in tennis training strategies vary over time, as the main influencing factors for tennis players also change accordingly. Therefore, the proposed model only addresses the psychological health status prediction of some tennis players. Further discussions should consider their current situation for predictions at other periods. (2) The AI-assisted RNN analysis technology used in this prediction model is only applied to tennis training, and its applicability to other sports and athletes needs further analysis.

## Data availability statement

Data will be made available on request.

## Ethics statement

The studies involving human participants were reviewed and approved by College Of Physical Education, China West Normal University Ethics Committee (Approval Number: 2022.193053). The participants provided their written informed consent to participate in this study. All methods were performed in accordance with relevant guidelines and regulations.

## CRediT authorship contribution statement

**Yan Du:** Writing – original draft, Software, Formal analysis, Data curation, Conceptualization. **Yujia Xia:** Software, Resources, Methodology, Investigation. **Lili Wang:** Visualization, Validation, Supervision. **Tiantian Zhang:** Validation, Resources, Methodology. **Linlin Ju:** Writing – review & editing, Visualization, Validation, Supervision.

## Declaration of competing interest

The authors declare that they have no known competing financial interests or personal relationships that could have appeared to influence the work reported in this paper.
