# Supplementary material for: The influence of psychological change factors of tennis training strategy using optimized recurrent neural network and artificial intelligence
Source: Heliyon. 2024 Jun 19;10(13):e33273. doi: 10.1016/j.heliyon.2024.e33273 (PMC11255447; doi:10.1016/j.heliyon.2024.e33273)
Supplement: Multimedia component 1 [file mmc1.docx]

Dear esteemed tennis players,

Hello!

We are conducting a study on “The Influence of Psychological Change Factors of Tennis Training Strategy Using Optimized Recurrent Neural Network and Artificial Intelligence" Thank you for taking the time out of your busy schedule to participate in this questionnaire. This research aims to understand and compare the physical and psychological changes of college tennis players before and after the optimized recurrent neural network-assisted training, to provide scientific evidence for the improvement of future training methods. Please answer the following questions truthfully according to your actual situation. All data will be kept strictly confidential and used only for academic research purposes.

1. Are you currently using an artificial intelligence-assisted system for tennis training?

A. Yes, currently using B. No, not yet using

2. How do you feel about your level of focus on the training content in your recent training sessions?

A. Very high B. High C. Moderate D. Low E. Very low

3. Do you think the current training plan is tailored to your individual characteristics and needs?

A. Completely personalized B. Basically meets personal needs C. Average, room for improvement D. Few considerations for individual characteristics E. Not personalized at all

4. How anxious do you feel before upcoming matches?

A. Very low B. Low C. Moderate D. High E. Very high

5. How helpful do you find the current training method in terms of tactical decision-making and action skill optimization?

A. Extremely helpful B. Quite helpful C. Average D. Slightly helpful E. Not helpful at all

6. How would you rate your ability for self-psychological regulation when facing training and match pressure?

A. Very strong B. Strong C. Moderate D. Weak E. Very weak

7. How satisfied are you with the physical and mental recovery and fatigue management after training and matches?

A. Very satisfied B. Satisfied C. Average D. Dissatisfied E. Very dissatisfied

8. Compared to the past, has your confidence increased?

A. Significantly increased B. Slightly increased C. Remained the same D. Slightly decreased E. Significantly decreased

9. How do you think the current training method affects your ability to execute tactics and adapt to changes on the field?

A. Greatly improved execution B. Improved execution to some extent C. No significant impact D. Slightly decreased execution to some extent E. Greatly decreased execution

10. If you have used an artificial intelligence-assisted system for training, how do you think it has affected your psychological state?

A. Significantly improved psychological state B. Improved psychological state somewhat, but not significantly C. No noticeable change in psychological state D. To some extent, affected psychological state, but not positively E. Significantly worsened psychological state

Thank you very much for patiently completing this questionnaire. Your valuable experience and feedback are crucial to our research. We will anonymize your data and rigorously apply it to the study to provide scientific evidence for optimizing training strategies for college tennis players and improving training effectiveness. In order to ensure the accuracy and effectiveness of the study, if you have any questions or need further communication during this questionnaire survey, please feel free to contact us at any time. Once again, thank you for your support and contribution to this research. We wish you excellent results in your future training and matches!

Sincerely!
